# Some of the Causes of Neuralgia

**Published:** 1895-10

**Authors:** 


### Some of the Causes of Neuralgia.

Recent investigations have brought out the fact that there are many severe cases of neuralgia caused by abnormal conditions of the nasal passages. In several instances there has been found enlargement of the bony structure of hard lumps of diseased tissue pressing against certain nerves and causing the most excruciating pain. Removal of these has resulted in complete cure, although there have been returns of the growths after the first operation. Persons who habitually suffer from pains in the head should have their conditions carefully diagnosed. Long continued suffering not infrequently brings about protracted and incurable mental and nervous disorders.

